# PLK1 phosphorylates RhoGDI1 and promotes cancer cell migration and invasion

**DOI:** 10.1186/s12935-024-03254-z

**Published:** 2024-02-14

**Authors:** Jeewon Lim, Yo Sep Hwang, Hyang Ran Yoon, Jiyun Yoo, Suk Ran Yoon, Haiyoung Jung, Hee Jun Cho, Hee Gu Lee

**Affiliations:** 1 Immunotherapy Research Center, Korea Research Institute of Bioscience and Biotechnology, Daejeon, 34141 Republic of Korea; 2 Department of Biomolecular Science, University of Science and Technology (UST), Daejeon, 34113 Republic of Korea; 3 Division of Applied Life Science, Gyeongsang National University, Jinju, 52828 Republic of Korea

**Keywords:** PLK1, RhoGDI1, RhoA, Migration, Cancer

## Abstract

**Background:**

Rho guanine nucleotide dissociation inhibitor 1 (RhoGDI1) plays an important role in diverse cellular processes by regulating Rho guanosine triphosphate (GTP)ases activity. RhoGDI1 phosphorylation regulates the spatiotemporal activation of Rho GTPases during cell migration. In this study, we identified polo-like kinase 1 (PLK1) as a novel kinase of RhoGDI1 and investigated the molecular mechanism by which the interaction between RhoGDI1 and PLK1 regulates cancer cell migration.

**Methods:**

Immunoprecipitation, GST pull-down assay, and proximity ligation assay (PLA) were performed to analyze the interaction between RhoGDI1 and PLK1. In vitro kinase assay and immunoprecipitation were performed with Phospho-(Ser/Thr) antibody. We evaluated RhoA activation using RhoGTPases activity assay. Cell migration and invasion were analyzed by transwell assays.

**Results:**

GST pull-down assays and PLA showed that PLK1 directly interacted with RhoGDI1 in vitro and in vivo. Truncation mutagenesis revealed that aa 90-111 of RhoGDI1 are critical for interacting with PLK1. We also showed that PLK1 phosphorylated RhoGDI1 at Thr7 and Thr91, which induces cell motility. Overexpression of the GFP-tagged RhoGDI1 truncated mutant (aa 90-111) inhibited the interaction of PLK1 with RhoGDI1 and attenuated RhoA activation by PLK1. Furthermore, the overexpression of the RhoGDI1 truncated mutant reduced cancer cell migration and invasion in vitro and suppressed lung metastasis in vivo.

**Conclusions:**

Collectively, we demonstrate that the phosphorylation of RhoGDI1 by PLK1 promotes cancer cell migration and invasion through RhoA activation. This study connects the interaction between PLK1 and RhoGDI1 to the promotion of cancer cell behavior associated with malignant progression, thereby providing opportunities for cancer therapeutic interventions.

**Supplementary Information:**

The online version contains supplementary material available at 10.1186/s12935-024-03254-z.

## Background

Cancer is the leading cause of premature death worldwide [1]. Most deaths in cancer patients are caused by metastasis accompanied by malignant tumor migration and invasion to enter nearby blood and lymphatic vessels and reach distant organs [2–5]. The Rho guanosine triphosphate (GTP)ase family controls essential cellular processes. It is involved in the migration and invasion of hepatocellular carcinoma, colorectal cancer, and breast cancer cells [6–10]. Rho GTPases function as molecular switches, cycling between an inactive state bound to guanosine diphosphate in the cytosol and an active state bound to GTP in the membrane. This cycle is tightly regulated by three types of proteins: guanine nucleotide exchange factors (GEFs), which convert guanosine diphosphate (GDP) to GTP; GTPase-activating proteins, which hydrolyze GTP to GDP; and Rho guanine nucleotide dissociation inhibitors (RhoGDIs), which bind to the most inactive Rho GTPases [11–14]. Activated Rho GTPases interact with their specific effector molecules and subsequently transduce downstream signaling cascades that regulate various physiological and pathophysiological cellular processes [15, 16]. Dysregulation of Rho GTPase activity is associated with malignant phenotypes of cancer cells.

RhoGDIs comprise RhoGDI1, RhoGDI2, and RhoGDI3 in mammals. The steady state of guanosine diphosphate-bound Rho GTPases is associated with cytosolic RhoGDIs [17, 18]. When Rho GTPases dissociate from RhoGDIs, they insert into the plasma membrane and are activated by GEFs. After completing their functions, RhoGDIs reassociate with Rho GTPases, causing their release from the plasma membrane and recycling them in the cytoplasm [19]. The dissociation of RhoGDI1 from Rho GTPases is regulated by interactions with specific proteins [16, 20, 21]. Phosphorylation of RhoGDI1 by protein kinases, such as tyrosine-protein kinase Src, p21-activated kinase 1, and serine/threonine kinase SAD-A, also decreases its affinity for Rho GTPases, promoting their subsequent activation [22–24]. In contrast, protein tyrosine phosphatase-PEST bound to β8 integrin dephosphorylates RhoGDI1, which sequesters inactive Rac1 and Cdc42 in the cytosol [25]. Protein phosphatase 1B also dephosphorylates RhoGDI1 and suppresses epidermal growth factor-induced activation of RhoA, Rac1, and Cdc42 [9]. These reports suggest that reversible phosphorylation of RhoGDI1 by protein kinases and phosphatases regulates the activity of Rho GTPases.

Polo-like kinase 1 (PLK1) is a serine/threonine protein kinase that modulates the biochemical properties of specific proteins via phosphorylation and subsequent modifications [26]. PLK1 is a key regulator of cell cycle progression, including mitosis and cytokinesis [27]. PLK1 also regulates the migration and invasion of various cancer cell lines, including colorectal, prostate, and lung cancer cells [28–30]. Although many studies have suggested a role for PLK1 in mitosis and cytokinesis, the precise mechanism of action of PLK1 in cell migration has been poorly studied. Using a proteomic approach, we previously identified PLK1 as a RhoGDI1-binding candidate protein [9]. In this study, we investigated the molecular mechanisms by which PLK1 regulates cell migration. Our results demonstrated that PLK1 activates RhoA by directly binding and phosphorylating RhoGDI1 at Thr7 and Thr91, promoting cell migration and invasion.

## Materials and methods

### Plasmids and reagents

Human RhoGDI1 and PLK1 cDNA were purchased from Origene (GenBank accession: NM_004309) and ABM (GenBank accession: BC014846). PCR-amplified Flag-RhoGDI1 and HA-PLK1 cloned into pCDNA3.1(+). Substitution mutants of RhoGDI1 and truncated mutants of RhoGDI1 were synthesized by service of Bioneer and then cloned into pCDNA3.1(+), pEGFP-N2, pET-28a(+), pGEX-4 T-1 or pCDH-CMV-MCS-EF1-Puro-GFP. Volasertib (BI 6727), a PLK1 inhibitor, was obtained from Selleckchem (S2235).

### Cell culture

293 T, HeLa, MDA-MB-468, and Hs578T cells were purchased from the ATCC. Cells were subcultured every 3 days in DMEM (Welgene, LM 001-05) with 10% fetal bovine serum and 1% antibiotics (Gibco, 11570486) at 37 °C, 5% CO2 in a humidified atmosphere.

### Cell transfection and lentivirus infection

Cells were cultured until 70–80% confluent, then transfection was performed using Lipofectamine 3000 (Invitrogen) with the manufacturer’s protocol. After incubation for 24–48 h at 37 °C, the cells were subjected to analyze. GFP-RhoGDI1 aa 90-111 stable HeLa cells were generated by infecting cells with lentiviruses expressing the respective recombinant RNAs and followed by 4 μg/ml puromycin selection for 5 passages.

### RNA interference

siRNA-based depletion was accomplished using AccuTarget Negative Control siRNA (siNC) and two different PLK1 siRNAs (siPLK1 #1: 5′-GUUCUUUACUUCUGGCUAUdTdT-3′, siPLK1 #2: 5′-GUUCGGGUGUGGGUUCUACAdTdT-3′) that were obtained from Bioneer. Transfection of 50 μM siRNA was performed with Lipofectamine RNAiMAX (Invitrogen) using the manufacturer’s protocol.

### Immunoprecipitation and western blot analysis

Cells were harvested in ice-cold RIPA buffer (100 mM Tris–HCl pH 7.6, 50 mM NaCl, 50 mM β-glycerophosphate, 50 mM NaF, 0.1 mM Na_3_VO_4_, 0.5% NP-40, 0.5% sodium deoxycholate and 5 mM EDTA) with protease inhibitor cocktail. Quantified protein lysates were incubated with anti-Flag (Sigma, F1804) and anti-HA (ABM, G036) at 4 °C overnight, followed by agarose beads were incubated for 2 h. Total protein lysates and immunoprecipitates were subjected to immunoblotting with anti-Flag-HRP (Sigma, A8592), anti-HA-HRP (Roche, 12013819001), anti-PLK1 (Cell Signaling Technology, 4513), anti-PLK1 (Santa Cruz Biotechnology, sc-17783), anti-RhoGDI1 (Santa Cruz Biotechnology, sc-373724), anti-RhoGDI1 (invitrogen, 51-1000Z), anti-GST (Santa Cruz Biotechnology, sc-138), anti-His (Santa Cruz Biotechnology, sc-8036), anti-Phospho-(Ser/Thr) (Cell Signaling Technology, 9631), anti-RhoA (Santa Cruz Biotechnology, sc-418), anti-Rac1 (Millipore, 05-389), anti-Cdc42 (BD Bioscience, 610929) and β-Actin (Santa Cruz Biotechnology, sc-47778).

### GST pull-down assay

His-fused PLK1, GST-fused wild-type (wt) RhoGDI1, and GST-fused RhoGDI1 truncation mutants were expressed in BL21 (DE3) competent cells. Lysates containing recombinant RhoGDI1 protein were mixed with GSH-beads (GE Healthcare, 17-0756-01) overnight at 4 °C. GST-RhoGDI1 bound beads were washed and incubated with His-PLK1 expression lysate at 4 °C for overnight. The samples were analyzed by western blotting.

### In vitro kinase assay

Recombinant wt or mutant RhoGDI1 were expressed in BL21 (DE3) and bound with Ni-NTA or GSH beads. Bead binding proteins were mixed with active His-PLK1 (3804-KS, R&D Systems) and 200 μM ATP in a kinase buffer (9802, Cell Signaling Technology). These mixtures were incubated at 30 °C for 1 h. The reactions were terminated using 2X SDS sample buffer. Samples were separated using SDS-PAGE and analyzed with western blot.

### RhoGTPases activity assay

Rho assay reagent (Millipore, 14-383) and Rac/Cdc42 assay reagent (Millipore, 14-325) were used for the RhoGTPases activity assay by the manufacturer's instructions. Cells were lysed with ice-cold MLB lysis buffer. Activation levels of RhoA, Rac1, and Cdc42 were assessed by pull-down assay using Rhotekin-agarose beads or PAK1-agarose beads and analyzed by immunoblotting using respective antibodies to RhoGTPases.

### Cell migration and invasion assays

Migration and invasion assays were evaluated with Corning Transwell polycarbonate membrane cell culture inserts (3422), as previously described [9].

### Cell proliferation assay

Cells were plated in a six-well plate at 1 × 10^5^ cells per well. After incubation for 1–3 days, cells were stained with 0.4% trypan blue. Viable cells were counted with Countess II FL Automated Cell Hemocytometer (Thermo Invitrogen).

### Proximity ligation assay (PLA)

PLA was performed with Duolink^®^ In Situ Red Starter Kit Mouse/Rabbit (Sigma Aldrich, DUO92101) by the manufacturer’s instructions. Briefly, HeLa cells were seeded in, fixed with 3.7% PFA for 30 min at RT, and blocked with blocking solution. Cells were incubated with indicated antibodies and followed by incubation with PLA probes. After probe incubation, cells were washed and incubated with ligation mixtures to form a closed circle. Next, amplification mixtures were added to cells for Rolling Circle Amplification (RCA) and hybridization of fluorescently labeled oligonucleotides to RCA. A mounting medium with DAPI was added to washed cells. PLA Signals from cells were imaged by a confocal microscope.

### Peptide synthesis and cell penetration assay

N-terminal FITC-labeled control cell-penetrating peptide (RRRRRRRR) and RhoGDI1 aa 90-111 peptide (RRRRRRRRLTGDLESFKKQSFVLKEGVEYR) were synthesized by Abclon. The purity of the peptides was guaranteed > 95% by the manufacturer’s HPLC and mass spectrometry. Cells were treated with 20 μM peptides for 2 h at 37 °C and washed three times with PBS. After fixing with 3.7% paraformaldehyde, cells were stained with 300 nM DAPI and analyzed using the confocal microscope.

### Confocal microscope and fluorescence imaging

Cellular images were visualized with a confocal microscope (LSM 800, Zeiss). The excitation/emission wavelengths were set to 590 nm/617 nm for Alexa Fluor 594, 499 nm/520 nm for Alexa Fluor 488, and 359 nm/457 nm for DAPI. Confocal cell images were analyzed using the ZEN 2.5 software.

### In vivo lung metastasis experiments

The experimental protocols were approved by the KRIBB Institutional Animal Care and Use Committee (KRIBB-AEC-23261), and all of the mice were housed under consistent temperature (22–25℃) and humidity (40–50%) in a specific pathogen-free (SFP) room. For the in vivo lung metastasis assay, 6–8 week old female BALB/c nude mice (purchased from Dae Han Bio Link Co., Ltd., Eumseong-gun, Korea) were randomly divided into two groups, 2 × 10^6^ HeLa-GFP or HeLa-GFP RhoGDI1 aa90-111 cells were intravenously injected into each mouse. 16 weeks after injection, the mice were euthanized, and their lungs were removed and subjected to hematoxylin and eosin (H&E) staining. The number of metastatic nodules was counted per lung section under the light microscope.

### Statistics

Experiments were conducted at least three times independently, and one representative data from experiments is shown in figure legend. Quantitative data were compared using Student's t-test and presented as means ± standard deviations. P < 0.05 was considered significant.

## Results

### PLK1 interacts with RhoGDI1 and phosphorylates it at Thr7 and Thr91

**Fig. 1:**
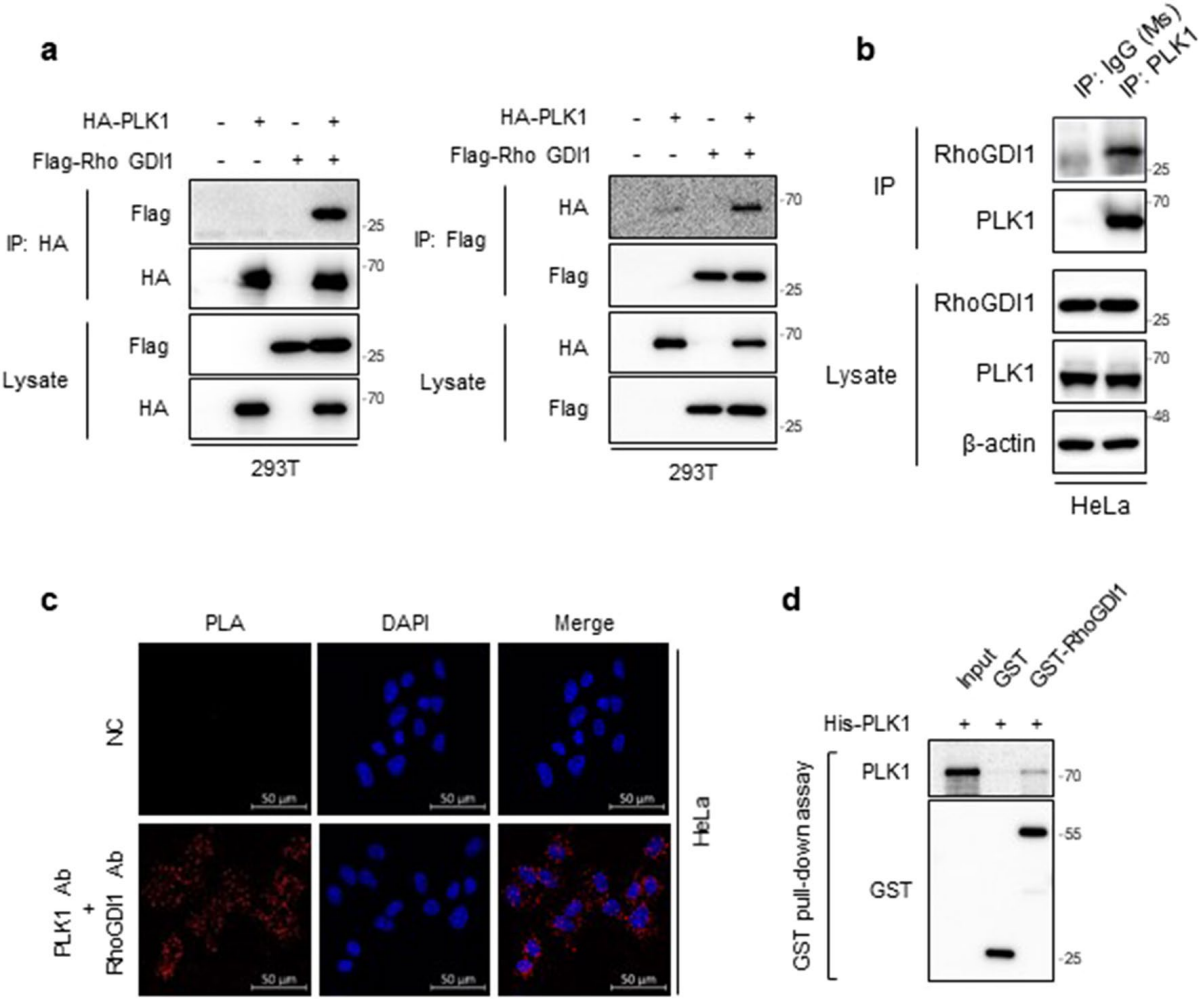
PLK1 directly binds to RhoGDI1 in vitro and in vivo. **a** 293 T cells were co-transfected with HA-tagged PLK1 and Flag-tagged RhoGDI1. Cell lysates were immunoprecipitated with HA (left) or Flag antibody (right). Immunoprecipitates and total lysates were immunoblotted with HA and Flag antibodies. **b** Cell lysates from HeLa were immunoprecipitated with IgG (as control) or PLK1 antibodies, and immunoblotted with PLK1 or RhoGDI1 antibodies. **c** Proximity ligation assay (PLA) was performed with PLK1 and RhoGDI1 antibodies in HeLa cells. Representative fluorescence images show blue (DAPI) staining nuclei and red dots (PLA signals). Scale bars: 50 μm. **d** His-tagged PLK1 and GST-tagged RhoGDI1 were expressed in *E.coli*. GST pull-down assay was performed with His-PLK1 expression lysates and purified GST-RhoGDI1. The GST pull-down products were immunoblotted with PLK1 and GST antibodies

Using a proteomic approach, we previously found that PLK1 is a candidate protein that binds to RhoGDI1 [9]. To confirm the interaction between these two proteins, we transfected Flag-RhoGDI1 and HA-PLK1 cells and performed a co-immunoprecipitation analysis. Flag-RhoGDI1 was detected in the HA-PLK1 immune complexes, and HA-PLK1 was found in the Flag-RhoGDI1 immune complexes (Fig. 1a). RhoGDI2 was not detected in the PLK1 immune complexes (Additional file 1: Fig. S1), indicating that PLK1 interacts with RhoGDI1, but not RhoGDI2. To investigate whether endogenous PLK1 and RhoGDI1 also interact with each other in HeLa cells, endogenous PLK1 was immunoprecipitated with an anti-PLK1 antibody. As expected, endogenous RhoGDI1 was observed in PLK1 immune complexes from HeLa cells (Fig. 1b). We also performed PLA that visualizes direct protein–protein interactions in cells [31]. PLA signals were detected in HeLa cells after the addition of specific antibodies against PLK1 and RhoGDI1 (Fig. 1c). Moreover, GST pull-down assays using GST-RhoGDI1 and His-PLK1 showed PLK1 binds to RhoGDI1 in vitro (Fig. 1d). The results suggest that PLK1 binds directly to RhoGDI1 in vitro and in vivo.

**Fig. 2:**
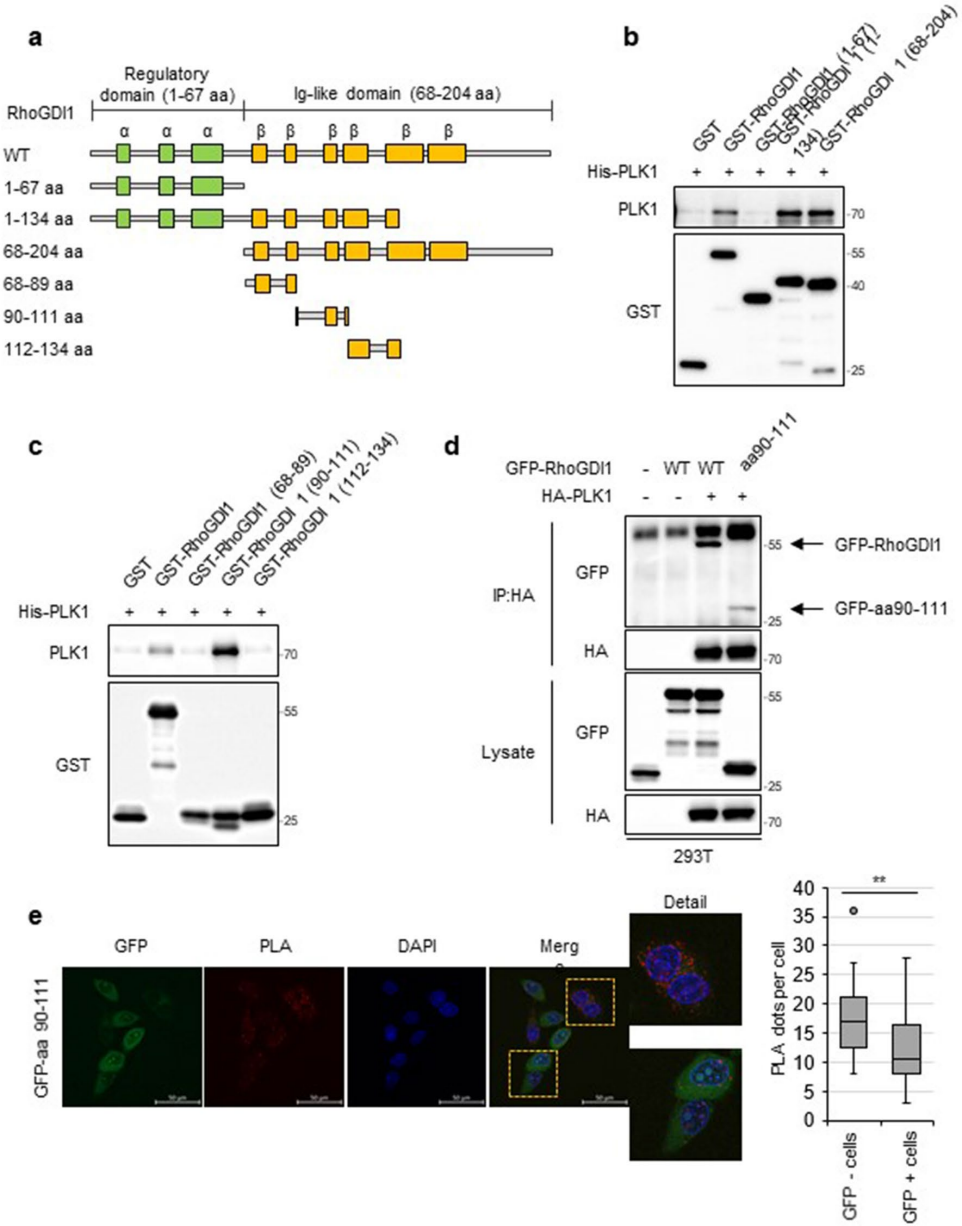
Mapping the region of RhoGDI1 for the interaction with PLK1. **a** Schematic representation of wild-type RhoGDI1 and truncation mutants of RhoGDI1 (1-67, 1-134, 68-204, 68-89, 90-111, 112-134 amino acids). **b**, **c** GST pull-down assay was performed with His-PLK1 expression lysates and purified GST-tagged wt RhoGDI1 or truncation mutants. The GST pull-down products were immunoblotted with PLK1 and GST antibodies. **d** 293 T cells were co-transfected with HA-tagged PLK1 and GFP-tagged RhoGDI1 wt or mutant (aa 90-111). Cell lysates were immunoprecipitated with a HA antibody. Immunoprecipitates and total lysates were immunoblotted with HA and GFP antibodies. **e** The proximity ligation assay was performed with PLK1 and RhoGDI1 antibodies in HeLa cells transfected with GFP-RhoGDI1 mutant (aa 90-111). Representative images (left) display blue (DAPI) staining nuclei and red dots (PLA signals). Scale bars: 50 μm. Quantitative data plots of PLA (right) show PLA dots per GFP-RhoGDI1 aa 90-111 positive or negative cells. **P < 0.01

Next, we aimed to identify the region of RhoGDI1 responsible for its interaction with PLK1. To this end, we bacterially expressed and purified GST-fused WT RhoGDI1, three GST-fused RhoGDI1 truncation mutants (Fig. 2a), and His-fused PLK1. GST pull-down assays revealed that His-PLK1 was pulled down along with GST-RhoGDI1 and two GST-RhoGDI1 truncation mutants consisting of aa 1-134 and aa 68-201 but not with GST alone, and a GST-RhoGDI1 truncation mutant consisting of aa 1-67, suggesting that the region of RhoGDI1 aa 68-134 is important for its interaction with PLK1 (Fig. 2b). To clearly identify the binding region of RhoGDI1 for the interaction with PLK1, we constructed three additional truncation mutants of RhoGDI1 aa 68-134 (Fig. 2a). RhoGDI1 aa 90-111 mutant bound to PLK1, while two mutants encompassing aa 68-89 and aa 112-134 region of RhoGDI1 did not (Fig. 2c). GFP-RhoGDI1 aa 90-111 mutant interacted with HA-PLK1 in HEK293T cells (Fig. 2d). GFP-RhoGDI1 aa 90-111-transfected cells showed significantly reduced PLA signals (red dots) compared to untransfected cells (Fig. 2e), indicating that GFP-RhoGDI1 aa 90-111 disrupted the interaction between these two proteins. These data suggest that the aa 90-111 of RhoGDI1 is indispensable for its binding to PLK1.

**Fig. 3:**
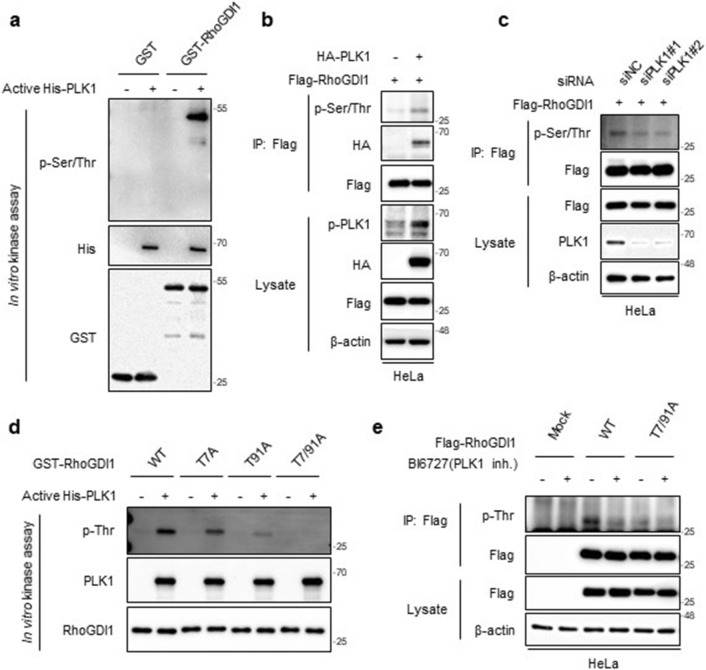
PLK1 phosphorylates RhoGDI1 at Thr7/91 residue in vitro and in vivo. **a** Purified GST-RhoGDI1 was subjected to in vitro kinase assay with( +) or without(-) active His-PLK1. The assay products were immunoblotted with the indicated antibodies. **b** Flag-RhoGDI1 was transfected with or without HA-PLK1 into HeLa cells. Cell lysates were immunoprecipitated with Flag antibody. Immunoprecipitates and total lysates were immunoblotted with the indicated antibodies. **c** HeLa cells were co-transfected Flag-RhoGDI1 with the control siRNA or two PLK1 siRNAs. Cell lysates were analyzed by Immunoprecipitation and immunoblotting using the indicated antibodies. **d** Purified His-RhoGDI1 wt and threonine-to-alanine-substituted mutants (T7A, T91A and T7/91A) were subjected to in vitro kinase assay with (+) or without (−) active His-PLK1. The assay products were immunoblotted with the indicated antibodies. **e** HeLa cells were transfected with Flag-RhoGDI1 wt or T7/91A and then treated with 200 nM of BI6727 (PLK1 inhibitor) for 4 h. Cell lysates were immunoprecipitated with Flag antibody. Immunoprecipitates and total lysates were immunoblotted with the indicated antibodies

As PLK1 is a serine/threonine kinase [26], we investigated whether PLK1 phosphorylates RhoGDI1. Bacterially purified GST-RhoGDI1 was reacted with commercially available active His-PLK1. In vitro kinase assay showed that active PLK1 phosphorylated GST-RhoGDI1 (Fig. 3a). To verify whether PLK1 phosphorylates RhoGDI1 in mammalian cells, HeLa cells were transfected with Flag-RhoGDI1 with mock or HA-PLK1. Immunoprecipitation and WB analysis revealed that PLK1 overexpression significantly increased RhoGDI1 phosphorylation compared to mock vector expression (Fig. 3b). Moreover, siRNA-mediated PLK1 depletion markedly reduced RhoGDI1 phosphorylation (Fig. 3c).

The motif D/E-X-S/T-φ-X-D/E (X, any amino acid; φ, a hydrophobic amino acid) has been reported as a consensus phosphorylation sequence by Plk1 [32]. Threonine residues in RhoGDI1 corresponding to the consensus motif were replaced with alanine residues (T7A, T91A, and T7/91A). Bacterially purified His-RhoGDI1 T7A, T91A, and T7/91A mutants were incubated with active His-PLK1. Threonine phosphorylation of RhoGDI1 by PLK1 was slightly reduced by T7A mutation and moderately suppressed by T91A mutation. However, the T7/91A mutation entirely disrupted the phosphorylation of RhoGDI1 by PLK1 (Fig. 3d). To investigate whether PLK1 phosphorylates RhoGDI1 at the Thr7 and Thr91 residues in vivo, HeLa cells were transfected with Flag-RhoGDI1 WT or the T7/91A mutant and then treated with DMSO as vehicle control or PLK1 inhibitor (BI 6727). Threonine phosphorylation was detected in WT RhoGDI1-transfected cells, and pharmaceutical inhibition of PLK1 reduced this phosphorylation. However, Threonine phosphorylation of RhoGDI1 was not detected in the presence or absence of BI 6727 in T7/91A-transfecting cells (Fig. 3e). Collectively, these data indicate that PLK1 phosphorylates RhoGDI1 at both Thr7 and Thr91 residues.

### PLK1-mediated phosphorylation of RhoGDI1 activates RhoA and promotes cancer cell migration and invasion

**Fig. 4:**
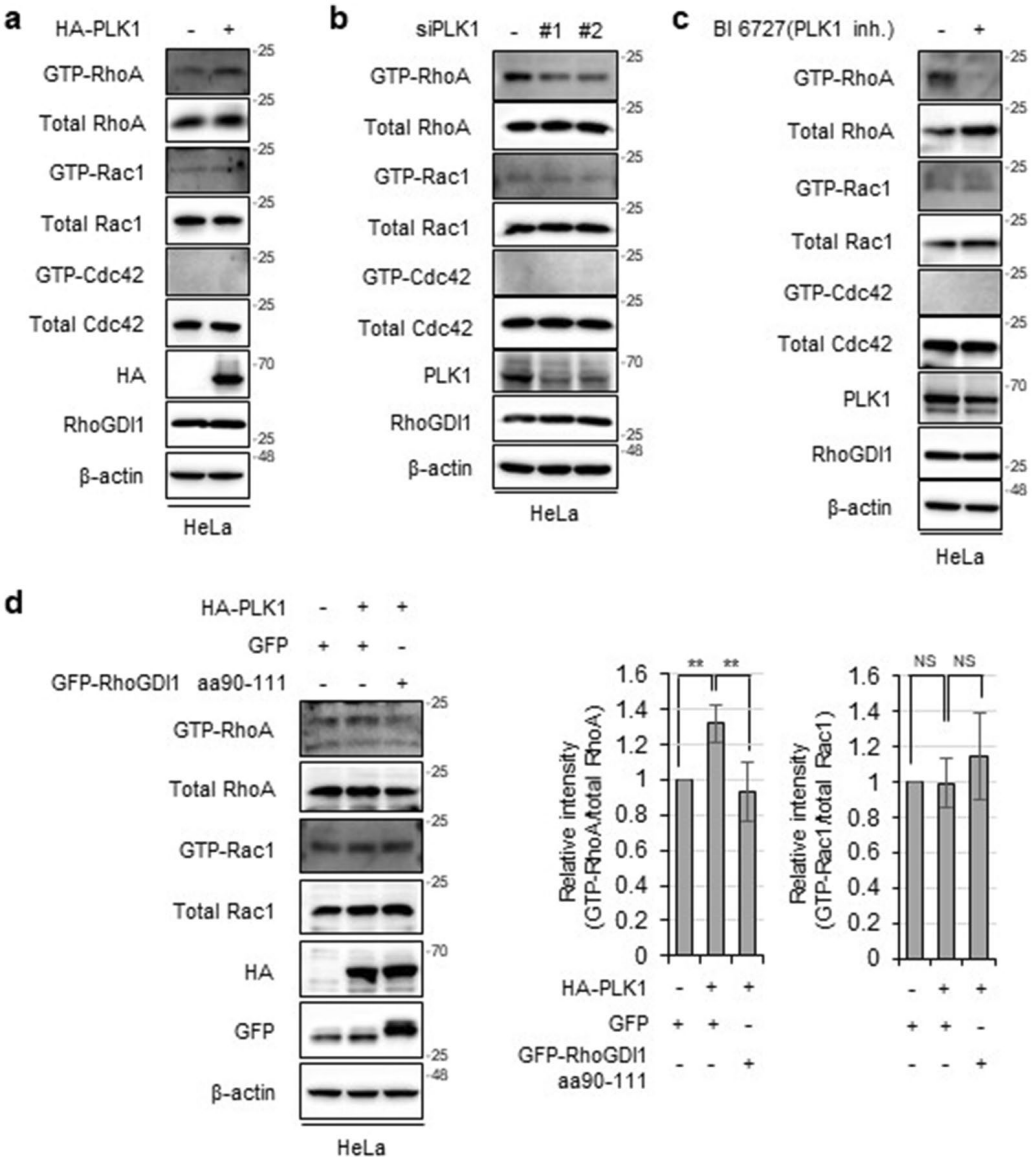
PLK1 promotes RhoA activation by association with RhoGDI1. **a** HeLa cells were transfected with mock or HA-PLK1. A pull-down assay was performed using Rhotekin-agarose beads to detect active RhoA and PAK1-agarose beads to detect active Rac1/Cdc42 as described in Materials and Methods **b** HeLa cells were transfected with control siRNA or two PLK1 siRNAs. Cell lysates were subjected to pull-down assay. **c** Cells were treated with 200 nM of BI 6727 for 4 h. Cell lysates were subjected to pull-down assay. **d** HeLa cells were co-transfected with HA-PLK1 along with GFP or GFP-RhoGDI1 aa 90-111. Cell lysates were subjected to pull-down assay (left panel). Relative intensity graphs show mean ± S.D. (n = 3) for GTP-RhoA/total RhoA or GTP-Rac1/total Rac1 (right panel). **P < 0.01

The phosphorylation of RhoGDI1 at different sites is a pivotal pathway for regulating Rho GTPase activity [33]. As we identified novel phosphorylation of RhoGDI1 by PLK1, we aimed to verify whether PLK1 affects the activity of Rho GTPases. GST pull-down assays were performed using the GST-rhotekin-binding domain for RhoA activity and the GST-Pak1 binding domain for Rac1/Cdc42 activity. The level of active-RhoA was elevated in PLK1-overexpressing cells compared to that in control cells. However, PLK1 did not affect the level of active-Rac1 or active-Cdc42 (Fig. 4a). Moreover, inhibition of PLK1 by siRNAs or BI 6727 significantly attenuated the level of active-RhoA but not active-Rac1 and active-Cdc42 (Fig. 4b and c).

To elucidate whether PLK1-induced RhoA activation was related to the interaction of PLK1 with RhoGDI1, we utilized the GFP-RhoGDI1 aa 90–111 mutant that binds to PLK1 and abrogates the interaction between PLK1 and endogenous RhoGDI1 (Fig. 4d). As expected, PLK1 overexpression enhanced RhoA activity. However, this increase was reversed by the GFP-RhoGDI1 aa 90–111 mutant (Fig. 4d). These results indicated that the interaction between PLK1 and RhoGDI1 was responsible for PLK1-mediated RhoA activation.

Since the activation of RhoA is required for cancer cell migration [34] and PLK1 enhances RhoA activity through the interaction with RhoGDI1 (Fig. 4), we investigated whether PLK1 could affect the migration and invasion of cancer cells. HeLa cells were treated with DMSO (vehicle control) or the PLK1-inhibitor BI 6727. Transwell migration and invasion assays revealed that treatment with BI 6727 significantly reduced the motility and invasiveness of HeLa cells compared with DMSO-treated HeLa cells (Additional file 1: Fig. S2a and b), suggesting that PLK1 activity is critical for cancer cell migration and invasion. To verify whether PLK1-mediated cell migration and invasion are associated with RhoGDI1 interaction, we co-transfected HA-PLK1 with the GFP-RhoGDI1 aa 90–111 mutant into HeLa cells and performed transwell migration and invasion assays. The results demonstrate that PLK1 overexpression enhanced cell migration and invasion. However, overexpression of the GFP-RhoGDI1 aa 90-111 mutant reversed these increases induced by PLK1 overexpression (Additional file 1: Fig. S2c and d). Since PLK1 overexpression enhances cell growth [35], we determined whether the interaction between PLK1 and RhoGDI1 also affected cell proliferation of HeLa cells co-transfected with HA-PLK1 and the GFP-RhoGDI1 aa 90-111 mutant. Although PLK1-expressing cells showed enhanced growth compared to control cells, the growth rate of cells expressing the RhoGDI1 mutant did not affect PLK1-mediated cell proliferation (Additional file 1: Fig. S2e). To evaluate whether PLK1-mediated phosphorylation of RhoGDI1 regulated cell motility, we performed migration and invasion assays using HeLa cells transfected with HA-PLK1 or/and Flag-RhoGDI1 WT or T7/91A mutant. PLK1 overexpression enhanced cell migration and invasion when co-transfected with RhoGDI1 WT but not RhoGDI1 T7/91A (Additional file 1: Fig. S3a and b). These data suggest that PLK1-mediated phosphorylation of RhoGDI1 at Thr7 and Thr91 residues is required for cell migration and invasion.

**Fig. 5:**
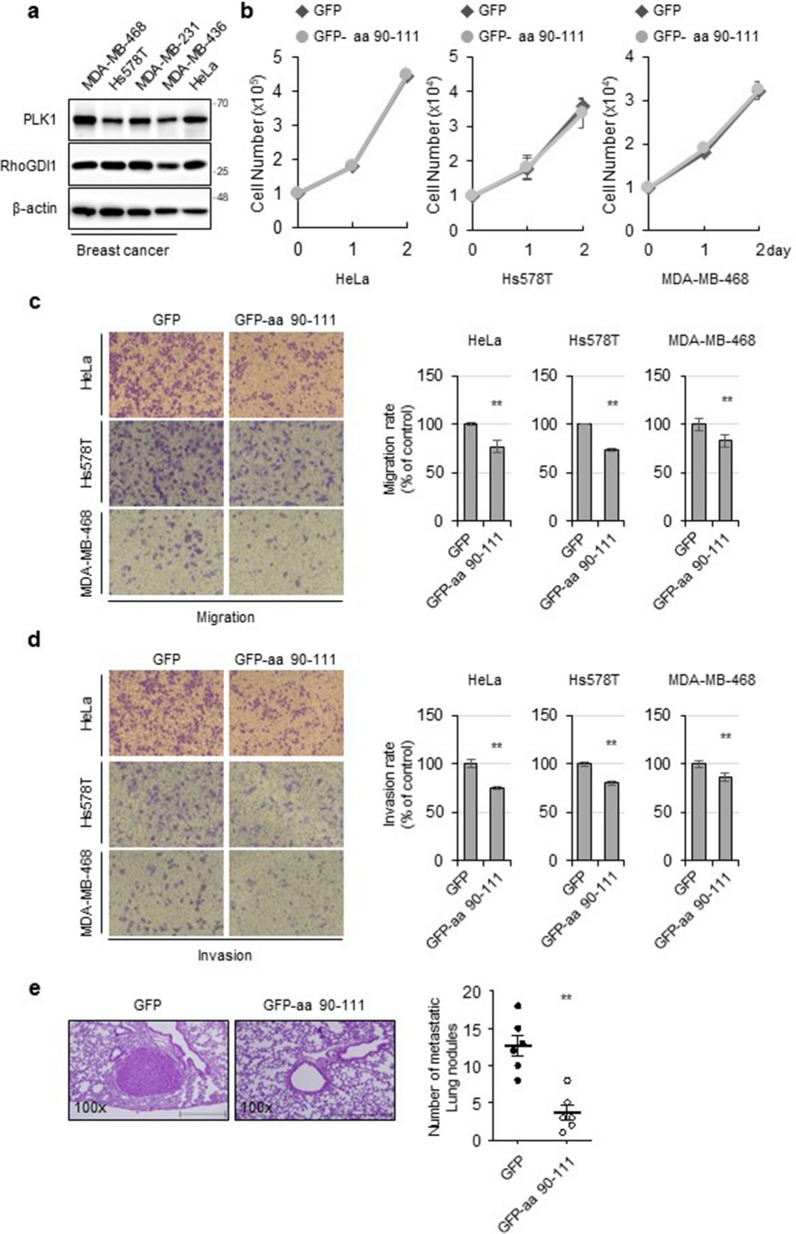
Interaction between PLK1 and RhoGDI1 is required for the migration and invasion of cancer cells. **a** Western blot analysis of PLK1 and RhoGDI1 expression in human cancer cell lines. **b** Indicated cells were transfected with GFP or GFP-RhoGDI1 aa 90–111. Cell proliferation was assessed by counting the viable cells after trypan blue staining at each day point. **c**, **d** Indicated cells were transfected with GFP or GFP-RhoGDI1 aa 90–111. 24 h after transfection, cells were serum-starved overnight and then subjected to migration **c** and invasion **d** assay as described in Materials and Methods. Representative images of migrating or invading cells stained with crystal violet were displayed (left). The relative percentages of migrating or invading cells were quantified as described in Materials and Methods (right). Quantitative data display the mean ± SD (n = 3). **e** 2 × 10.^6^ HeLa cells that overexpress GFP (control) or GFP-RhoGDI1 aa 90-111 were injected via the tail vein of female BALB/c nude mice (n = 6 per group). Representative image of H&E stained lung tissue from HeLa-GFP (control) or HeLa-GFP-RhoGDI1 aa 90-111 (left). The number of metastatic nodules was counted per lung section and displayed in the scatter plots (right). Quantitative data display the mean ± SD (n = 6). Scale bar = 500 μm. **P < 0.01

To confirm the effect of the interaction between endogenous PLK1 and RhoGDI1 on cancer cell migration and invasion, we first assessed their expression in human breast cancer and HeLa cervical cancer cells. WB analysis showed that PLK1 and RhoGDI1 were expressed in all breast cancer cell lines and HeLa cells (Fig. 5a). Next, we investigated the effect of the GFP-RhoGDI1 aa 90-111 mutant on cancer cell proliferation. HeLa, Hs578T, and MDA-MB-468 cells were transfected with the mock or GFP-RhoGDI1 aa 90-111-expressing vector. As expected, overexpression of the GFP-RhoGDI1 aa 90-111 mutant did not affect the growth rate of the HeLa, Hs578T, or MDA-MB-468 cells (Fig. 5b). However, cell migration and invasion were attenuated in cells expressing GFP-RhoGDI1 aa 90-111 compared with those in control vector-expressing cells (Fig. 5c and d). To further assess the function of RhoGDI1 aa 90-111 mutant on distant metastasis in vivo, exogenous GFP-RhoGDI1 aa 90-111 was stably overexpressed into HeLa cells, which is subjected to evaluate the cell motility. Consistent with transiently transfected cells, cell migration and invasion in vitro were reduced in HeLa cells overexpressing GFP-RhoGDI1 aa 90-111 compared with those in GFP-overexpressing control cells (Additional file 1: Fig. S4a and b). GFP-RhoGDI1 aa 90-111-overexpressing cells and GFP-overexpressing cells were intravenously injected into female nude mice, respectively. The lung metastases in mice were observed by H&E staining 16 weeks after injection. The metastatic nodules of lung sections derived from HeLa-GFP-RhoGDI1 aa 90-111 cells were significantly fewer than those derived from HeLa-GFP cells (Fig. 5e). These results demonstrate that the interaction between RhoGDI1 and PLK1 promotes cell migration and invasion as well as distant metastasis.

### Synthetic peptide derived from RhoGDI1 attenuates cancer cell migration and invasion

**Fig. 6:**
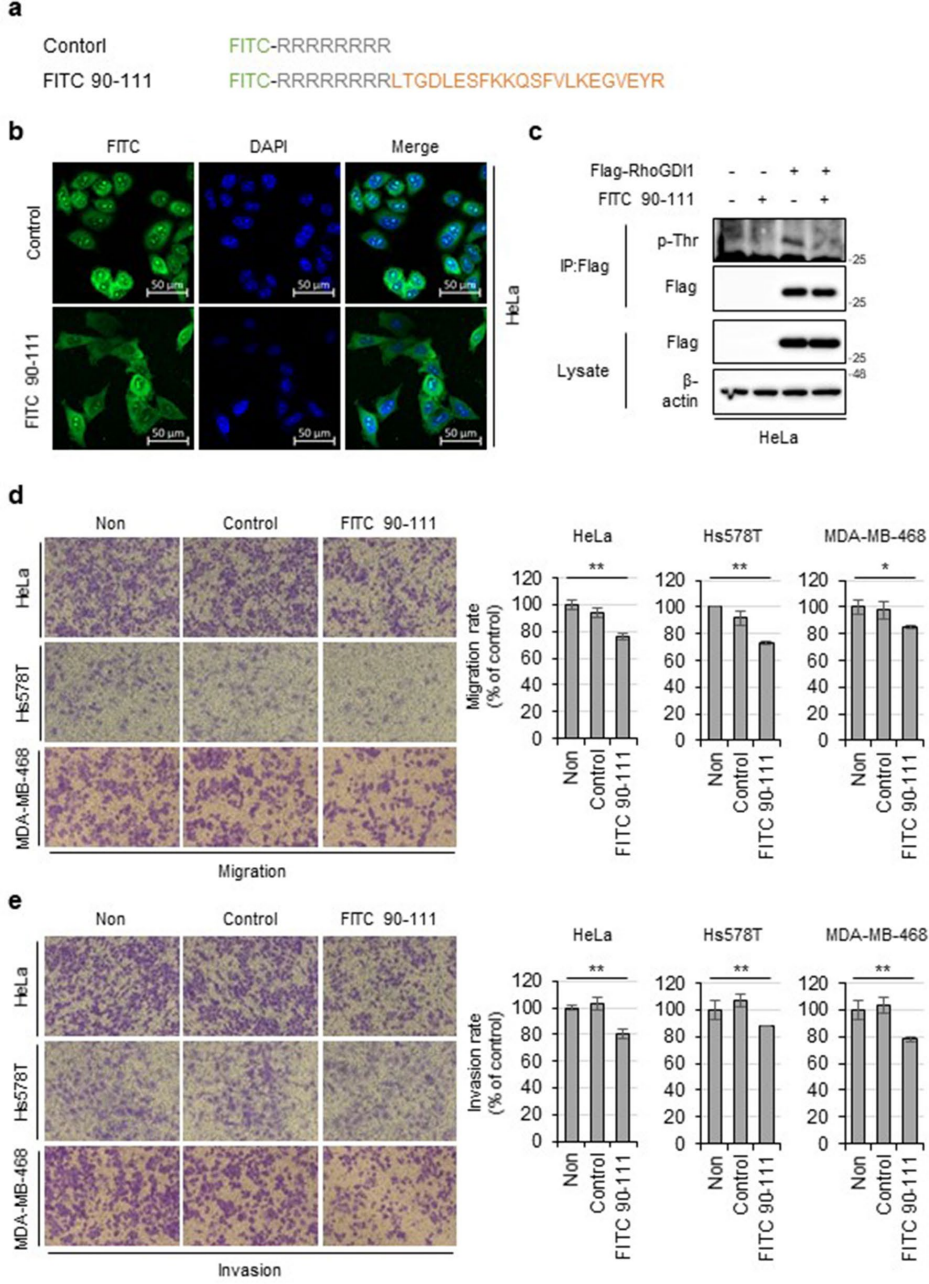
The synthetic peptide containing RhoGDI1 aa 90-111 inhibits cancer cell migration and invasion. **a** FITC-labeled synthetic peptide sequence. **b** Cellular uptake of FITC-labeled peptides by HeLa cells. Scale bar = 50 μm. Data are representative of three independent experiments. **c** HeLa cells were transfected with Flag-RhoGDI1 and treated with 20 μM peptides for 12 h. The lysates were immunoprecipitated with Flag antibody. Immunoprecipitates and total lysates were immunoblotted with the indicated antibodies. **d**, **e** Indicated cells were treated with 20 μM peptides and subjected to migration **(d)** and Invasion **(e)** assay. Representative images of migrating or invading cells (left). The relative percentages of migrating or invading cells (right). Quantitative data display the mean ± S.D. (n = 3). *P < 0.05; **P < 0.01

Next, we investigated whether a peptide containing the PLK1 binding sequence of RhoGDI1 (aa 90-111) could reduce RhoGDI1 phosphorylation and suppress cancer cell migration and invasion. First, we constructed a FITC-labeled peptide containing RhoGDI1 aa 90-111 conjugated to the cell-penetrating sequence (FITC 90-111) and its control peptide (Fig. 6a). These peptides were treated for 4 h and internalized into HeLa cells (Fig. 6b). Next, to verify the effect of the RhoGDI1 peptide on the PLK1-mediated phosphorylation of RhoGDI1, HeLa cells were transfected with Flag-RhoGDI1 and then incubated with control or FITC 90-111 peptides. WB analysis revealed that the FITC 90-111 peptide markedly attenuated the phosphorylation of Flag-RhoGDI1 compared to the control peptide (Fig. 6c). Importantly, the FITC 90-111 peptide significantly suppressed the migration and invasion of HeLa, Hs578T, and MDA-MB-468 cells (Fig. 6d and e). These results indicate that the interaction with PLK1 via RhoGDI1 aa 90-111 plays a crucial role in cancer cell migration and invasion by regulating RhoGDI1 phosphorylation.

## Discussion

PLK1 is overexpressed in various human cancers, including melanoma [36], prostate cancer [37], colorectal cancer [38], hepatocellular carcinoma [39], and breast cancer [40], and its expression is associated with enhanced cell proliferation and poor prognosis in patients with cancer [41]. PLK1 is a serine/threonine kinase involved in multiple steps of cell cycle progression [27]. Recent studies have suggested that PLK1 regulates the motility and invasiveness of several cancer cells [28–30]. PLK1 is overexpressed in colorectal cancer and promotes the migration and invasion of colon cancer cells [28]. PLK1 inhibition suppresses the migration of A549 lung adenocarcinoma cells by decreasing MMP2 and VEGFA expression [29]. PLK1 also induces epithelial-to-mesenchymal transition and promotes cell motility by activating the CRAF/ERK signaling pathway in prostate cancer cells [30]. Although several studies have provided evidence for the pro-migratory activity of PLK1, its precise mechanism remains to be thoroughly investigated.

The present study established a novel mechanism by which PLK1 regulates the migration and invasion of cancer cells. We identified that PLK1 interacts with RhoGDI1 and demonstrated that the aa 90-111 region of RhoGDI1 is essential for its interaction with PLK1. Abrogation of the interaction between PLK1 and RhoGDI1 by overexpression of GFP-RhoGDI1 aa 90-111 attenuated the migration and invasion promoted by PLK1 overexpression. However, inhibition of the interaction between these two proteins did not affect PLK1-mediated cell proliferation. Furthermore, a synthetic peptide corresponding to aa 90-111 derived from RhoGDI1 suppressed cancer cell migration and invasion. These data indicated that interaction between PLK1 and RhoGDI1 was required for PLK1-mediated cell migration, invasion and metastasis in vivo.

In the present study, we identified PLK1 as a novel kinase involved in RhoGDI1 phosphorylation. PLK1 directly phosphorylated RhoGDI1 at Thr7 and Thr91 in vitro. PLK1 overexpression increased the phosphorylation of RhoGDI1. In contrast, the inhibition of PLK1 activity suppressed its phosphorylation. PLK1 could phosphorylate RhoGDI1 but not RhoGDI2 which does not bind to PLK1 (Additional file 1: Fig. S5); this suggests that the interaction between PLK1 and RhoGDI1 is essential for phosphorylation. It has been known that several kinases can phosphorylate RhoGDI1 at Ser101, Ser174, and Tyr156 to regulate the spatiotemporal activity of Rho GTPases, which is critical for cell migration [22, 23]. Src-mediated phosphorylation of RhoGDI1 at Tyr156 promotes RhoGDI1 accumulation at membrane ruffling and local activation of RhoA, Rac1, and Cdc42 [22]. PAK1 phosphorylates RhoGDI1 at Ser101 and Ser174, resulting in the activation of Rac1. As PAK1 is a downstream kinase of Rac1 and Cdc42, the PAK1-mediated phosphorylation of RhoGDI1 causes positive feedback regulation of Rac1 activation [23]. These studies suggest that the phosphorylation of RhoGDI1 at different sites regulates the spatiotemporal activation of Rho GTPases, leading to cell motility. Consistent with this, our data show that PLK1 promotes cell migration and invasion by phosphorylating RhoGDI1 at Thr7 and Thr91 residues (Additional file 1: Fig. S3).

Our results showed that PLK1 overexpression increased RhoA activity, whereas PLK1 knockdown by siRNA and inhibition of PLK1 activity using a pharmacological inhibitor decreased RhoA activity. The abrogation of the interaction between PLK1 and RhoGDI1 by overexpression of GFP-RhoGDI1 aa 90-111 suppressed PLK1-mediated RhoA activation as well as the cell migration and invasion promoted by PLK1. Therefore, although we cannot exclude the possibility that PLK1 regulates cell migration and invasion via other signaling pathways, such as CRAF/ERK and STAT3 [29, 30], the pro-migratory effect of PLK1 may be, at least partially, attributable to the increased RhoA activity following its interaction with RhoGDI1. As RhoGDI1 phosphorylation promotes the dissociation of RhoGDI1 from specific Rho GTPases [23, 42–44], we investigated whether PLK1 regulates the displacement of RhoGDI1 from Rho GTPases. Interestingly, PLK1 overexpression caused the dissociation of RhoA, Rac1, and Cdc42 from RhoGDI1 (Additional file 1: Fig. S6). In contrast, only RhoA is activated by PLK1. These results suggest that PLK1-mediated dissociation of RhoGDI1 is necessary but insufficient for Rho GTPase activation. The PLK1-mediated activation of Rho GTPases may also require other regulators, such as specific RhoGEFs. Similarly, our previous study demonstrated that ephrinB1 reverse signaling causes the dissociation of RhoA and Rac1 but promotes only RhoA activation [8]. The connector enhancer of KSR1 (CNK1) links the interaction between ephrinB1 and p115RhoGEF to promote the activation of RhoA [45], suggesting that additional factors, such as CNK1/p115RhoGEF, are required for RhoA activation by ephrinB1 reverse signaling. Indeed, PLK1 phosphorylates MyoGEF and allows it to localize at the central spindle, where it activates RhoA [46, 47]. A recent study showed that PLK1 phosphorylates CYK4, and a spatial gradient of phosphorylated CYK4 around the central spindle promotes local activation of RhoA by interacting with ECT2 on the adjacent plasma membrane during cytokinesis [48]. Future studies need to elucidate the precise mechanism by which additional factors, such as RhoGEFs, are involved in PLK1-mediated RhoA activation during cell migration.

## Conclusions

**Fig. 7:**
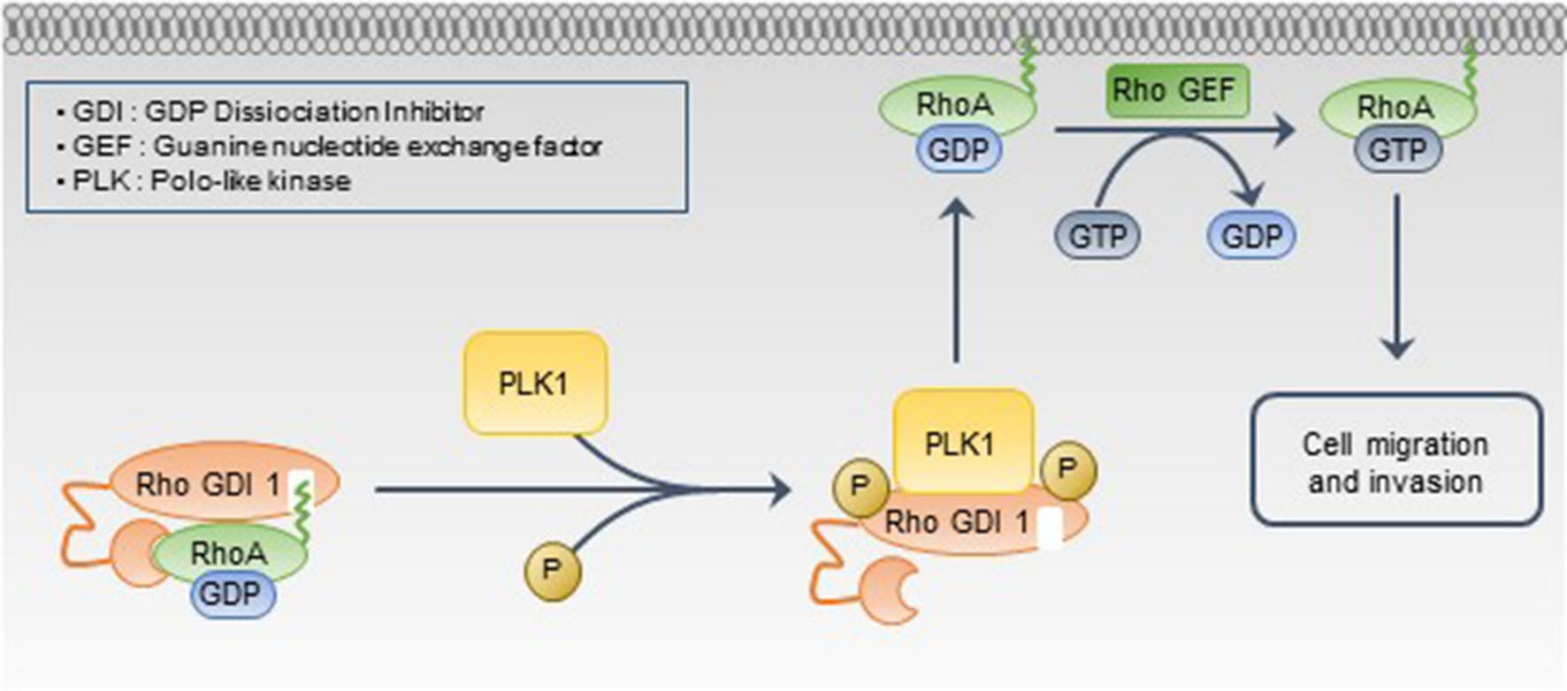
The proposed model to illustrate how PLK1 promotes cell migration and invasion

In the current study, we identified PLK1 as a novel kinase of RhoGDI1 and provided new mechanistic insight into the regulation by PLK1 of cancer cell migration and invasion via RhoA activation (Fig. 7). Our data suggest that inhibiting the PLK1/RhoGDI1 interaction using a synthetic peptide derived from RhoGDI1 may be a potential therapeutic approach for cancer treatment.

### Supplementary Information

**Additional file 1: Figure S1.** PLK1 binds to RhoGDI1 but not RhoGDI2. **a, b** 293T cells** (a)** and HeLa cells** (b)**were co-transfected HA-PLK1 with Flag-RhoGDI1 or Flag-RhoGDI2.Cell lysates were immunoprecipitated with HA antibody.Immunoprecipitates and total lysates were immunoblotted with HA and Flagantibodies. ** Figure S2** Interaction of RhoGDI1 with PLK1 is required for cell migration and invasion through Rho Aactivation. ** a,b**Transwell migration assay** (a)**and transwell invasion assay** (b)**were performed using HeLa cells treated with 50 nM of BI6727.Representative images of migrating or invading cells stained with crystal violet were displayed (left).The relative percentages of migrating or invading cells was quantified as described in Materials and Methods (right). **c-e**HeLa cells were co-trasnfected HA-PLK1 with GFP or GFP-RhoGDI1 aa 90-111.Transwell migration** (c)**and invasion** (d)**assay were performed using transfected cells.Representative images of migrating or invading cells (left).The relative percentages of migrating or invading cells (right).Quantitative data display the mean ± S.D. (n=3). **P<0.01. Cell proliferation was assessed by counting the viable cells after trypan blue staining at each day point **(e)**. ** Figure S3** PLK1-mediated phosphorylation of RhoGDI1 is required for cell migration and invasion. ** a,b** HeLa cells were trasnfected HA-PLK1 with Flag-RhoGDI1 WT or Flag-RhoGDI1T7/91A.Transwell migration **(a)** and invasion **(b)** assay were performed using co-transfected cells.Representative images of migrating or invading cells (left). The relative percentages of migrating or invading cells (right).Quantitative data display the mean ± S.D. (n=3). *P<0.05; **P<0.01. ** Figure S4** Inhibition of interaction with RhoGDI1 and PLK1 attenuates cell migration and invasion.** a,b** Transwell migration assay **(a) **and transwell invasion assay **(b) **were conducted with HeLa cells stably transfected with GFP o rGFP-RhoGDI1 aa 90-111. Representative images of migrating or invading cells (left). The relative percentages of migrating or invading cells (right). Quantitative data display the mean ± S.D. (n=3). **P<0.01. ** Figure S5 ** PLK1 phosphorylates RhoGDI1 but not RhoGDI2. Purified GST-RhoGDI1 or RhoGDI2 were incubated with active His-PLK1. The assay products were immunoblotted with the indicated antibodies. ** Figure S6 ** PLK1 promotes the dissociation RhoGTPases from RhoGDI1. HeLa cells were co-transfected Flag-RhoGDI1 with control vector or HA-PLK1. Cell lysates were immunoprecipitated with Flag antibody.

## Data Availability

The datasets generated during the current study are available from the corresponding author on reasonable request.
