# Supplementary material for: PLK1 phosphorylates RhoGDI1 and promotes cancer cell migration and invasion
Source: Cancer Cell Int. 2024 Feb 14;24:73. doi: 10.1186/s12935-024-03254-z (PMC10865702; doi:10.1186/s12935-024-03254-z)
Supplement: Supplementary file 1 — Additional file 1: Figure S1. PLK1 binds to RhoGDI1 but not RhoGDI2. a, b 293T cells (a) and HeLa cells (b)were co-transfected HA-PLK1 with Flag-RhoGDI1 or Flag-RhoGDI2.Cell lysates were immunoprecipitated with HA antibody.Immunoprecipitates and total lysates were immunoblotted with HA and Flagantibodies. Figure S2 Interaction of RhoGDI1 with PLK1 is required for cell migration and invasion through Rho Aactivation. a,bTranswell migration assay (a)and transwell invasion assay (b)were performed using HeLa cells treated with 50 nM of BI6727.Representative images of migrating or invading cells stained with crystal violet were displayed (left).The relative percentages of migrating or invading cells was quantified as described in Materials and Methods (right). c-eHeLa cells were co-trasnfected HA-PLK1 with GFP or GFP-RhoGDI1 aa 90-111.Transwell migration (c)and invasion (d)assay were performed using transfected cells.Representative images of migrating or invading cells (left).The relative percentages of migrating or invading cells (right).Quantitative data display the mean ± S.D. (n=3). **P<0.01. Cell proliferation was assessed by counting the viable cells after trypan blue staining at each day point (e). Figure S3 PLK1-mediated phosphorylation of RhoGDI1 is required for cell migration and invasion. a,b HeLa cells were trasnfected HA-PLK1 with Flag-RhoGDI1 WT or Flag-RhoGDI1T7/91A.Transwell migration (a) and invasion (b) assay were performed using co-transfected cells.Representative images of migrating or invading cells (left). The relative percentages of migrating or invading cells (right).Quantitative data display the mean ± S.D. (n=3). *P<0.05; **P<0.01. Figure S4 Inhibition of interaction with RhoGDI1 and PLK1 attenuates cell migration and invasion. a,b Transwell migration assay (a) and transwell invasion assay (b) were conducted with HeLa cells stably transfected with GFP o rGFP-RhoGDI1 aa 90-111. Representative images of migrating or invading cells (left). The relat [file 12935_2024_3254_MOESM1_ESM.pdf]

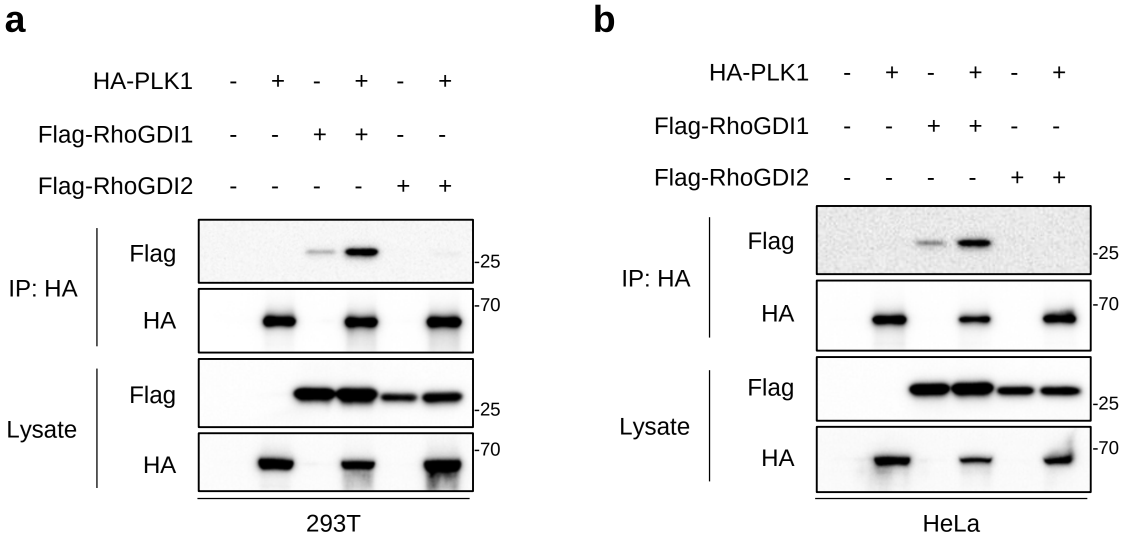

Fig. S1 PLK1 binds to RhoGDI1 but not RhoGDI2. **a, b** 293T cells **(a)** and HeLa cells **(b)** were co-transfected HA-PLK1 with Flag-RhoGDI1 or Flag-RhoGDI2. Cell lysates were immunoprecipitated with HA antibody. Immunoprecipitates and total lysates were immunoblotted with HA and Flag antibodies.

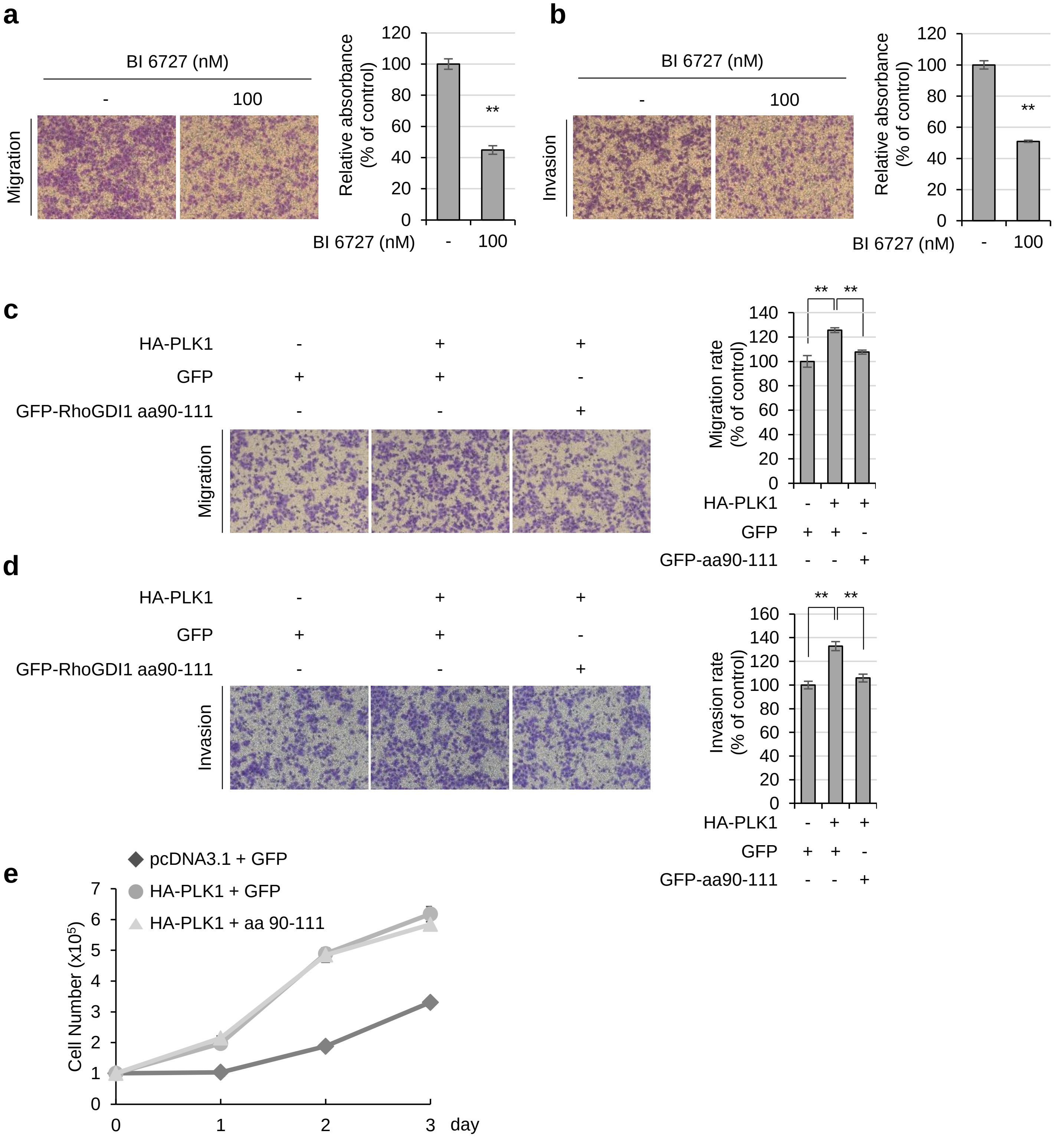

Fig. S2 Interaction of RhoGDI1 with PLK1 is required for cell migration and invasion through RhoA activation. **a, b** Transwell migration assay **(a)** and transwell invasion assay **(b)** were performed using HeLa cells treated with 50 nM of BI6727. Representative images of migrating or invading cells stained with crystal violet were displayed (left). The relative percentages of migrating or invading cells was quantified as described in Materials and Methods (right). **c-e** HeLa cells were co-trasnfected HA-PLK1 with GFP or GFP-RhoGDI1 aa 90-111. Transwell migration **(c)** and invasion **(d)** assay were performed using transfected cells. Representative images of migrating or invading cells (left). The relative percentages of migrating or invading cells (right). Quantitative data display the mean ± S.D. (n=3). **P<0.01. Cell proliferation was assessed by counting the viable cells after trypan blue staining at each day point **(e)**.

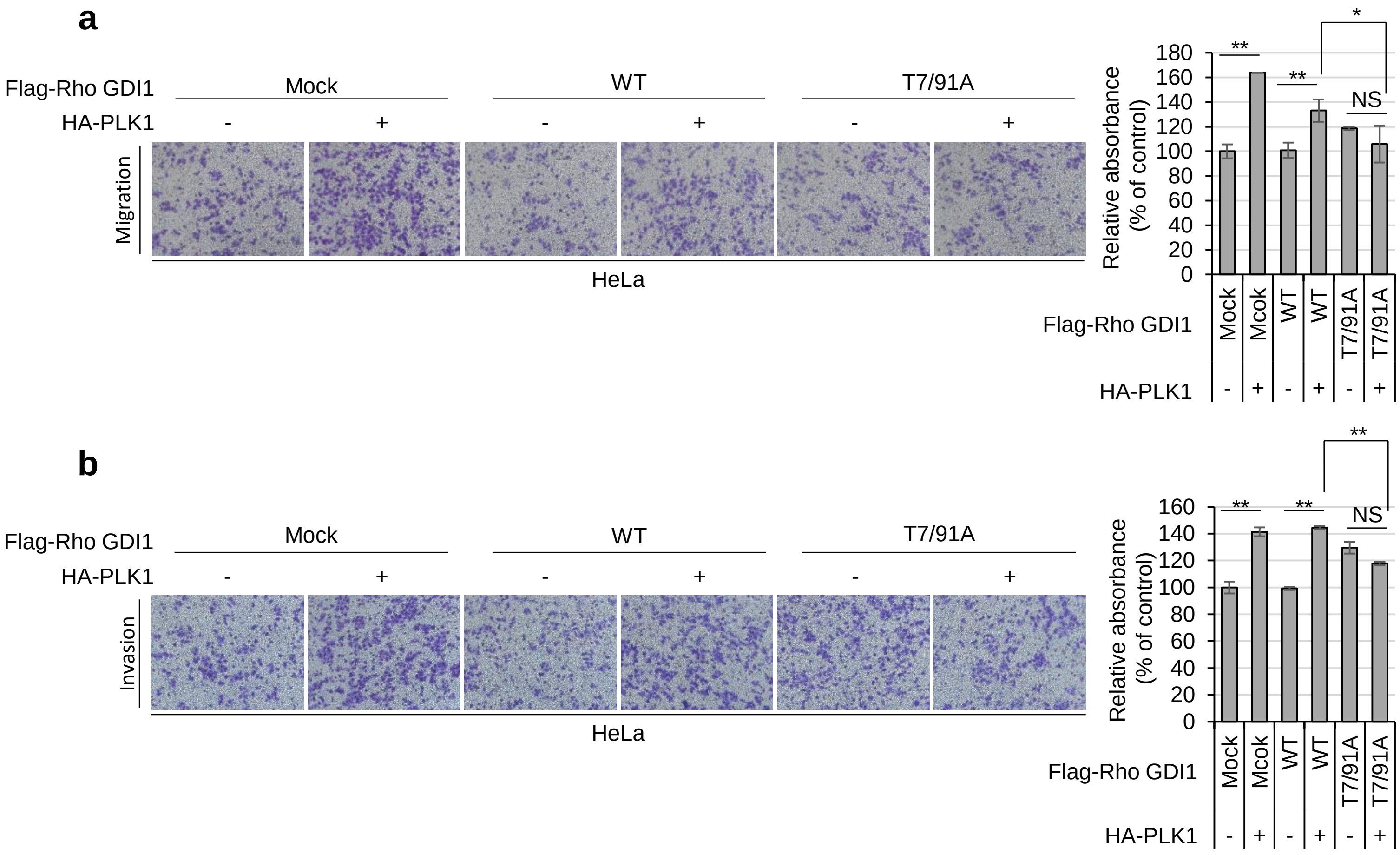

Fig. S3 PLK1-mediated phosphorylation of RhoGDI1 is required for cell migration and invasion. **a, b** HeLa cells were trasnfected HA-PLK1 with Flag-RhoGDI1 WT or Flag-RhoGDI1 T7/91A. Transwell migration **(a)** and invasion **(b)** assay were performed using co-transfected cells. Representative images of migrating or invading cells (left). The relative percentages of migrating or invading cells (right). Quantitative data display the mean ± S.D. (n=3). *P<0.05; **P<0.01.

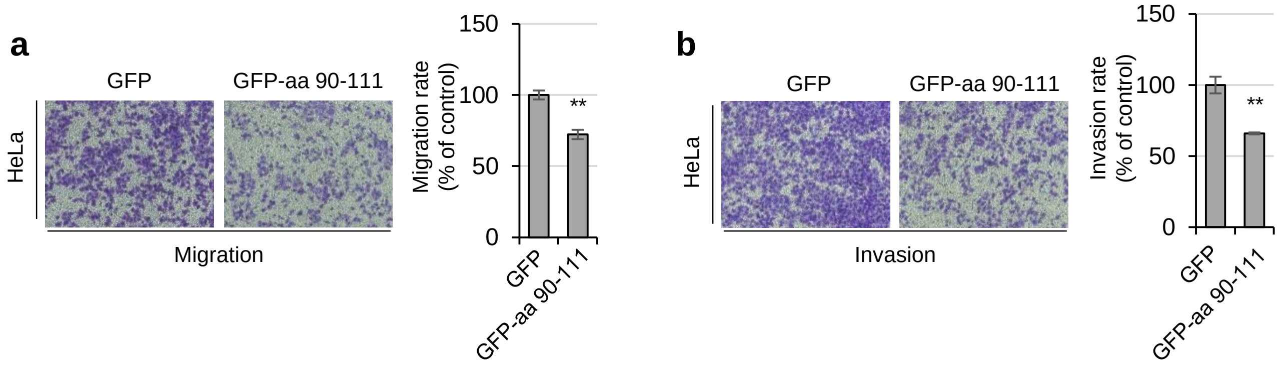

Fig. S4 Inhibition of interaction with RhoGDI1 and PLK1 attenuates cell migration and invasion. **a, b** Transwell migration assay **(a)** and transwell invasion assay **(b)** were conducted with HeLa cells stably transfected with GFP or GFP-RhoGDI1 aa 90-111. Representative images of migrating or invading cells (left). The relative percentages of migrating or invading cells (right). Quantitative data display the mean ± S.D. (n=3). **P<0.01.

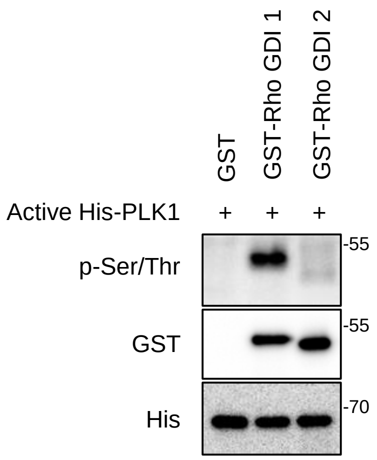

Fig. S5 PLK1 phosphorylates RhoGDI1 but not RhoGDI2. Purified GST-RhoGDI1 or RhoGDI2 were incubated with active His-PLK1. The assay products were immunoblotted with the indicated antibodies.

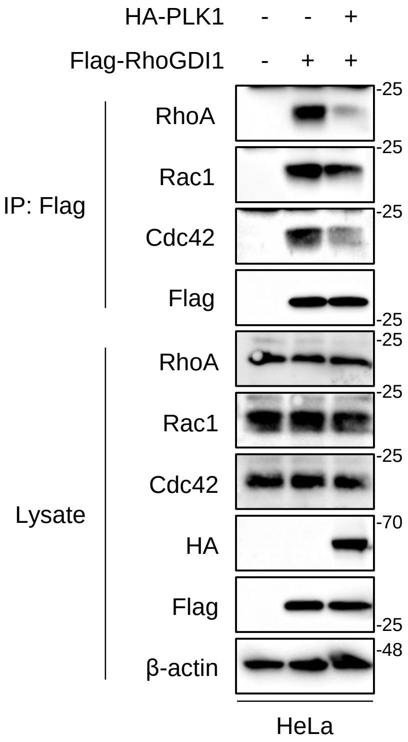

Fig. S6 PLK1 promotes the dissociation RhoGTPases from RhoGDI1. HeLa cells were co-transfected Flag-RhoGDI1 with control vector or HA-PLK1. Cell lysates were immunoprecipitated with Flag antibody.
